# Noninvasive Maternal–Fetal Hemodynamic Monitoring as A Predictor of Severe Preeclampsia in Low-Resource Settings: A Case Report

**DOI:** 10.1055/a-2689-2550

**Published:** 2025-09-05

**Authors:** Lilian Toledo-Jaldin, Richard Gomez, Litzi Lazo-Vega, Alison Larrea, Adolfo Vásquez, Wilson Ormachea-Orellana, Valquiria Miranda-Garrido, Colleen G. Julian

**Affiliations:** 1 Department of Obstetrics, Hospital Materno Infantil, La Paz, Bolivia; 2 Departments of Biomedical Informatics and Medicine, University of Colorado Anschutz Medical Campus, Aurora, Colorado

**Keywords:** preeclampsia, early detection, hemodynamics, low-resource settings

## Abstract

Preeclampsia is a multiorgan vascular disease complicating approximately 8.5 million pregnancies worldwide annually and is a leading cause of maternal and neonatal mortality. The impact is especially severe in Latin America, where maternal deaths attributable to preeclampsia are 2.5 times higher than in any other region. Bolivia is particularly affected due to economic and environmental challenges, including high altitude, which increases the risk of fetal growth restriction and hypertensive disorders of pregnancy. Early and accessible diagnostic tools are required to maximize patient care and improve reproductive outcomes in limited-resource settings. This report details a case from Bolivia of rapid-onset severe preeclampsia with liver rupture in the third trimester; the patient required multiple surgical interventions for recurrent liver bleeding and extended hospitalization in the intensive care unit (ICU). She delivered a preterm, growth-restricted infant with signs of acute hypoxia by emergency cesarean section. Notably, 2 weeks before ICU admission, abnormal uterine artery and maternal hemodynamic measurements were detected, without other signs of preeclampsia. The patient had previously been healthy and was considered low risk. Both mother and newborn survived. This case underscores the value of combining uterine artery Doppler with maternal hemodynamics to identify high-risk pregnancies early and prevent life-threatening complications.

Preeclampsia is one of the leading causes of maternal and perinatal mortality 1 2 and disproportionally affects resource-limited countries with limited resources where nearly all such deaths occur. 3 Bolivia is a low- to middle-income country with the third-highest maternal and infant mortality rates in the Western Hemisphere. 4 5 Among the several factors responsible for this vulnerability are limited access to or use of health care resources, socioeconomic limitations, and the fact that almost half of the Bolivian population lives at high altitudes (2500 m) where the incidence of preeclampsia and fetal growth restriction is three times higher than at lower altitudes, even under comparable health care conditions. 6 7 8 Critically needed are noninvasive and user-friendly tools for the early detection of high-risk pregnancies, such tools provide the opportunity for increased surveillance, careful timing of delivery, and the appropriate allocation of resources to minimize catastrophic outcomes.

Impaired placentation is indisputably central to the pathogenesis of early-onset preeclampsia, commonly defined as preeclampsia that develops before 34 weeks of pregnancy. In normal pregnancy, extravillous trophoblasts initiate extensive vascular remodeling of the spiral uterine spiral arteries 9 to facilitate uninterrupted blood flow to the uteroplacental circulation. Early placentation defects in preeclampsia include abnormalities in the development of the chorionic villi, shallow invasion of the endometrium by trophoblasts, insufficient remodeling of spiral arteries into low-resistance vessels, and, in turn, reduced uteroplacental blood flow. Placental hypoperfusion evokes excessive production and release of antiangiogenic and inflammatory factors, triggering widespread maternal endothelial dysfunction and the hallmark of preeclampsia. 10 Late-onset preeclampsia has been hypothesized to be due to placental hypoperfusion and dysfunction resulting not from insufficient placentation, but rather from impaired maternal cardiovascular function. 11

Maternal hemodynamic profiles differ between preeclamptic and normotensive pregnancy. During a healthy pregnancy, total uteroplacental blood flow and the proportion of maternal CO (cardiac output) directed to the uteroplacental circulation increases dramatically. These effects are due to increased maternal CO resulting from reduced systemic vascular resistance (SVR), greater blood volume, and a redistribution of blood flow to favor uteroplacental circulation. On the contrary, women who develop early-onset preeclampsia are reported to have elevated SVR, lower heart rate, as well as reduced stroke volume and CO. 12 13 14 On the contrary, pregnant women who develop late-onset preeclampsia have shown a higher stroke volume, CO, and heart rate with lower SVR compared with normotensive pregnant women. 12 13 14 Abnormal Dopplers of the uterine artery (UA) in the second and third trimesters are considered indicators of impaired placental function and an increased risk of obstetric complications in some, 15 16 but not all studies 17 18 indicating that these measurements may have limited specificity and sensitivity to predict adverse outcomes when considered in isolation.

Maternal central hemodynamic evaluations in the preclinical phases of preeclampsia can therefore provide critical information on the clinical trajectory, expand the temporal window to initiate treatment, and identify cases with a greater probability of severe adverse maternal–fetal outcomes. In resource-limited settings with minimal intensive care capacity for adults and newborns, initiating therapy as early as possible is particularly vital to minimize the probability of severe prematurity that requires advanced care and to prevent maternal death or other severe outcomes, including liver rupture.

Here, we report the case of a young, healthy mother who developed rapidly severe preeclampsia with hepatic rupture in the third trimester that was preceded by abnormal maternal uterine and central hemodynamic profiles but without traditional indications of preeclampsia.

## Case Report

**Fig. 1:**
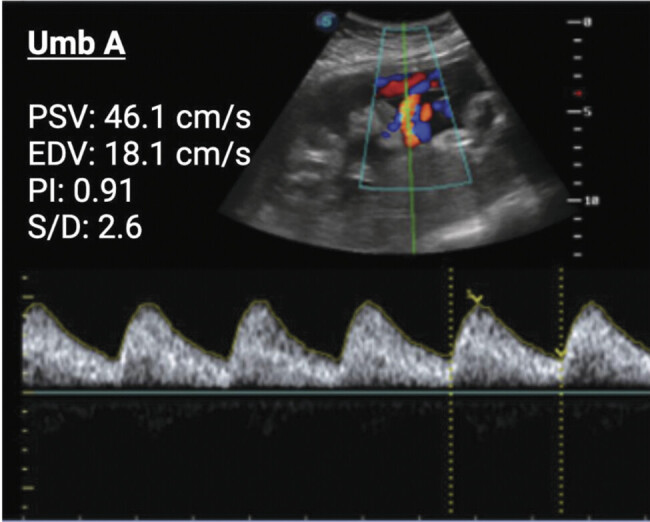
Doppler ultrasound study of fetal umbilical artery (Umb A) for the presented case at 29 weeks of gestation. Peak systolic velocity (PSV), end-diastolic velocity (EDV), pulsatility index (PI), and systolic-to-diastolic ratio (S/D) values were obtained for the Umb A.

**Fig. 2:**
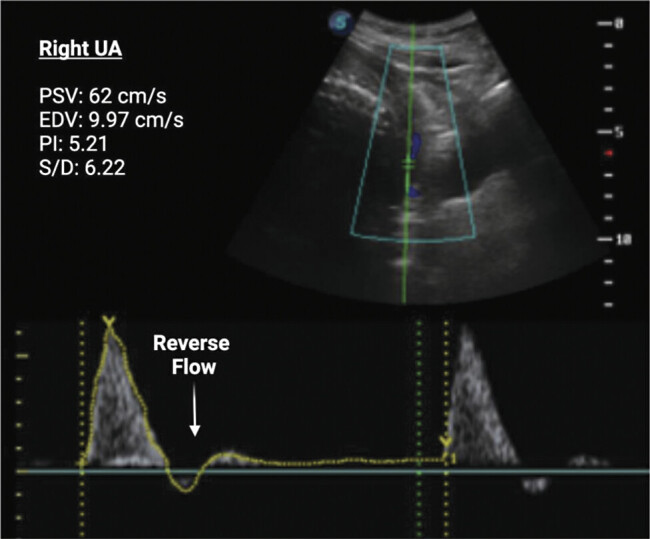
Doppler study of the maternal right uterine artery (UA) for the presented case at 29 weeks of pregnancy. Peak systolic velocity (PSV), end-diastolic velocity (EDV), pulsatility index (PI), and systolic-to-diastolic ratio (S/D) values were obtained for the right UA. A white arrow indicates the reverse flow in the flow trace.

A 33-year-old Hispanic patient 2G1P with no significant medical history, aside from a primiparous pregnancy that ended in a spontaneous abortion in the first trimester, presented to our obstetric unit for a regularly scheduled prenatal care visit at 29 weeks of pregnancy. On examination, she reported a moderate intensity headache and normal systemic blood pressure (110/84 mm Hg). Laboratory tests revealed a modest elevation of aspartate aminotransferase (AST, 51 U/L) and alanine aminotransferase (ALT, 95 U/L), without significant proteinuria (139 mg/24 h urine) or thrombocytopenia (platelet count, 356,000 mm ^3^ ). Obstetric ultrasound indicated severe restriction of fetal growth, with an estimated fetal weight of 1,010 g (percentile 0 Hadlock) and normal umbilical and middle cerebral artery pulsatility indices (PI) of 0.91 and 1.70, respectively ( Fig. 1 ). Doppler measurements of the ureteral artery were abnormal, with elevated PI on the right and left sides (5.2 and 3.9, respectively; > 95 ^th^ percentile) and reversed diastolic blood flow ( Fig. 2 ). Maternal central hemodynamic studies performed using USCOM showed low CO (2.7 L/min) and cardiac index (1.5 l/min/m ^2^ ), paralleled by elevated SVR, 2,905 dynes/s/cm 5 ) and SVR index (5,012 dynes/s/cm ^5^ /m ^2^ ; Table 1 ).

**Table 1:** Maternal central hemodynamic measurements at 29 weeks of gestation

Variable	Value
Peak flow velocity (m/s)	1.1
Mean pressure gradient (mm Hg)	1.9
Velocity time integral (cm)	21
Heart rate (bpm)	53
Minute distance (m/min)	11
Ejection time (%)	29
Flow time (ms)	334
Flow time, corrected (ms)	312
Systolic volume (cm ^3^ )	51
Systolic volume index (mL/m ^2^ )	29
Cardiac output (L/min)	2.7
Cardiac index (L/min/m ^2^ )	29
Systemic vascular resistance (ds cm ^−5^ )	2,905
Systemic vascular resistance index (ds cm ^−5^ m ^2^ )	5,012
Stroke volume variability (%)	55
Inotropy index (W/m ^2^ )	1.1
Potential kinetic ratio	54
Stroke work (mJ)	632
Cardiac power (W)	0.55

At 31.3 weeks of pregnancy, just before her next scheduled prenatal visit, the patient was admitted to the emergency room with severe headache, epigastric pain, severe hypertension (160/100 mm Hg), significant proteinuria (++ + ), elevated creatinine (1.2 mg/dL), thrombocytopenia (platelet count, 100,000 mm ^3^ ), and elevated transaminases (AST 634 U/L, ALT 631 U/L). Within 4 hours of admission, the patient developed hypovolemic shock for which she underwent a laparotomy and cesarean section to deliver a live female infant weighing 1,250 g. Liver packing, placement of a Bogota bag, cavity lavage, and installation of a Bakri balloon were performed in the event of uterine hypotonia. Placental histological reports revealed placental infarction and hemorrhage.

On the third postoperative day, the patient developed multiorgan dysfunction that included acute respiratory, hepatic, cardiovascular, and Akin III renal failure due to hypovolemic shock. At this time, the patient underwent a repeat laparotomy due to active hepatic bleeding with transoperative findings of hepatic laceration of segments V and VII. Ultimately, the patient underwent eight surgical procedures, one due to obstetric shock septic shock, for which a subtotal hysterectomy and a right adnexectomy were performed. During the surgical procedure, hepatic ischemia was observed in segments V to VIII. During hospitalization, laboratory examinations showed severe thrombocytopenia, hypertransaminasemia, hyperbilirubinemia, hyperazotemia, leukocytosis, and anemia due to excessive blood loss resulting from hemoperitoneal and surgical interventions. The patient's clinical course was further complicated by hospital-acquired pneumonia.

The patient received three sessions of renal replacement therapy with continuous venovenous hemofiltration and broad-spectrum antibiotic therapy according to the etiological agents identified in abdominal and bronchial secretion cultures. Due to the prolonged intubation time, a conventional tracheotomy was performed on day 14 of intubation, and parenteral nutrition was indicated. After 55 days in the intensive care unit, the patient and her infant were discharged in good condition.

## Discussion

We present a case of a previously healthy young woman who developed rapid-onset severe preeclampsia with hepatic rupture 2 weeks after a routine prenatal visit during which abnormal maternal systemic and UA hemodynamic measurements and fetal growth restriction were observed without any other indications of preeclampsia. Our observations suggest that noninvasive hemodynamic assessments, in combination with laboratory studies and standard obstetric ultrasound, may enable the identification of patients who later develop severe hypertensive emergencies at high altitudes.

Early diagnosis of preeclampsia allows the initiation of antihypertensive therapy before a rapid deterioration of clinical symptoms into a hypertensive emergency and before the development of severe maternal and fetal adverse outcomes. 19 If maternal hypertension cannot be controlled, iatrogenic preterm delivery can be required before 34 weeks of gestation, with associated significant perinatal risks, particularly in resource-limited settings. For the patient presented in this case study, we believe that if treatment had been started on the first observation of elevated SVR alongside reversed UA diastolic flow, the clinical course for this patient could have been less aggressive.

More broadly, we propose that hemodynamic-guided therapy may improve blood pressure management during pregnancy and, therefore, prevent poor outcomes among women at increased risk of developing preeclampsia at high altitudes. To support this approach, a retrospective study demonstrated that early antihypertensive therapy guided by maternal hemodynamics reduced the rate of severe maternal hypertension among women with an elevated risk of preeclampsia at low altitudes. 20 Clinical trials show that antihypertensive therapy to normalize maternal blood pressure reduces the risk of severe hypertension. 21 22 However, highlighting the need for close hemodynamic evaluation and concordant therapy adjustment, antihypertensive use, pronounced reduction in CO, or increased vascular resistance have been associated with reduced fetal growth and well-being. 20 23 24 25 Therefore, there is an ongoing debate about the appropriate blood pressure targets for women with mild to moderate hypertension during pregnancy.

## Conclusion

Future studies to systematically test the predictive value of maternal hemodynamic evaluations, alone or in combination with UA Dopplers, for preeclampsia are warranted and required before incorporating maternal hemodynamic evaluation for standard preeclampsia screening and guided therapies in Bolivia. We consider that the combined use of UA Dopplers and noninvasive measurement of maternal central hemodynamics may be a more powerful method to detect high-risk pregnancies with placental insufficiency and guide antihypertensive treatment at high altitudes. Preventive treatment in time and early diagnoses are necessary to prevent catastrophic adverse effects in pregnancy, particularly in low-resource settings with limited intensive care capacity to accommodate severe adverse maternal outcomes and premature newborns.

## Notes

**Corrigendum** : This article has been corrected as per the corrigendum published on September 18, 2025 (Doi: 10.1055/a-2698-8526).
