# Corrigendum: Noninvasive Maternal–Fetal Hemodynamic Monitoring as A Predictor of Severe Preeclampsia in Low-Resource Settings: A Case Report

**DOI:** 10.1055/a-2698-8526

**Published:** 2025-09-18

**Authors:** Lilian Toledo-Jaldin, Richard Gomez, Litzi Lazo-Vega, Alison Larrea, Adolfo Vásquez, Wilson Ormachea-Orellana, Valquiria Miranda-Garrido, Colleen G. Julian

**Affiliations:** 1 Department of Obstetrics, Hospital Materno Infantil, La Paz, Bolivia; 2 Departments of Biomedical Informatics and Medicine, University of Colorado Anschutz Medical Campus, Aurora, Colorado

It has been brought to the publisher's attention that the name of an author was listed incorrectly in an article published in *American Journal of Perinatology Reports* , Volume 15, Issue 3 on September 5, 2025 (doi:  ). The name was originally published as Valquiria Miranda-Girrado and has been corrected to Valquiria Miranda-Garrido. The correction has been made in the original article and correct author name also appears above.

